# Lessons to be learned from a missed case of Hamate fracture: a case report

**DOI:** 10.1186/1749-799X-5-64

**Published:** 2010-08-27

**Authors:** Vishal H Borse, James Hahnel, Adnan Faraj

**Affiliations:** 1 Vishal Borse, Room 346, Institute of Medical & Biological Engineering, School of Mechanical Engineering, University of Leeds, Leeds, LS2 9JT, UK; 2 James Hahnel, Department of Orthopaedics, Pinderfields General Hospital, Aberford road, Wakefield, WF1 4DQ, UK; 3 Adnan Faraj, Department of Orthopaedics, Airedale District General Hospital, Skipton road, Steeton, Keighley, BD20 6TD, UK

## Abstract

**Introduction:**

We report the case of a missed fracture through the body of the hamate bone, only detected on a later, mistakenly taken 30° oblique x-ray view. This case highlights some of the problems encountered with traditional x-ray views, and the need to consider oblique views as either standard procedure or as an adjunct where clinical suspicion remains high even in the presence of normal x-rays.

**Case presentation:**

A healthy 26-year-old Caucasian male fell whilst jogging, suffering a low velocity injury to his right hand. Initial accident and emergency examination and x-rays failed to demonstrate a fracture. At clinic, anteroposterior and carpal tunnel radiographs showed no fracture, however a mistakenly taken oblique x-ray revealed a displaced hamate body fracture.

**Conclusion:**

The authors believe that where a hamate fracture is suspected, an oblique x-ray view should be considered as part of the initial diagnostic investigations. Furthermore an oblique x-ray view is of particular use when clinical suspicion for hamate fracture remains high in the light of otherwise normal x-rays.

## Introduction

Hamate fractures are uncommon, particularly those involving the body of the hamate [1]. This case highlights some of the problems encountered with traditional x-ray views for identifying hamate fractures, and the need to consider oblique views as either standard procedure or as an adjunct where clinical suspicion remains high, even in the presence of normal x-rays.

## Case

A 26 year old Caucasian male tripped whilst jogging suffering a low velocity injury to his right hand. He fell hitting his metacarpophalangeal (MCP) joints against the corner of the road curb, with his fist clenched and his wrist in slight palmar flexion. He complained of immediate pain to the base of the middle and ring finger metacarpal bones of his right hand.

**Figure 1:**
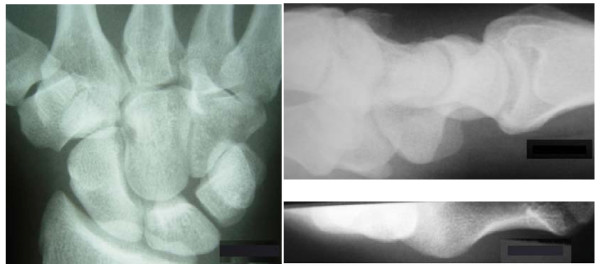
**Anteroposterior, lateral and carpal tunnel x-ray views (clockwise from left)**.

The patient presented to accident and emergency the same day where examination revealed bony tenderness and obvious bruising and swelling to the injured area, however x-rays failed to demonstrate a fracture (Figure 1).

**Figure 2:**
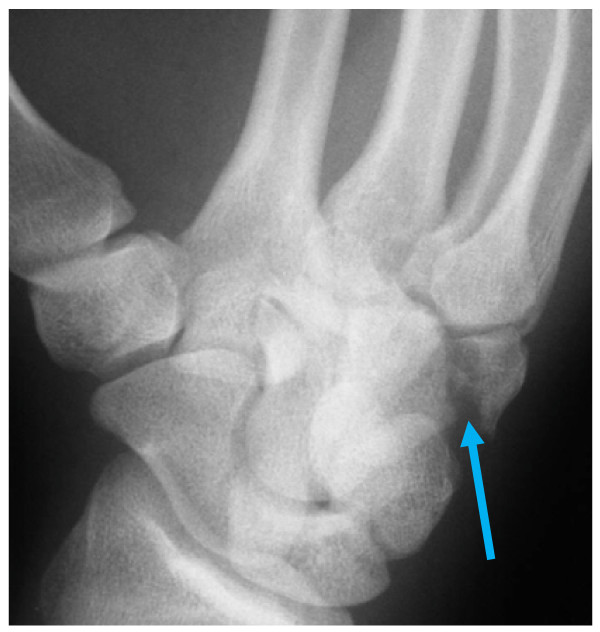
**Pronated oblique 30° x-ray view**. Blue arrow shows fracture site.

The patient's hand was not placed in plaster and he was referred to the orthopaedic outpatient clinic. Examination in clinic revealed bruising, swelling and bony tenderness to his 3^rd ^and 4^th ^MCP joints and due to the high index of suspicion, further anteroposterior (AP), lateral and carpal tunnel x-rays were requested. The AP and carpal tunnel radiographs showed no fracture, however an oblique x-ray was mistakenly taken instead of the requested lateral. This was an error on the part of the radiographer's. This oblique view revealed a displaced hamate body fracture (Figure 2).

**Figure 3:**
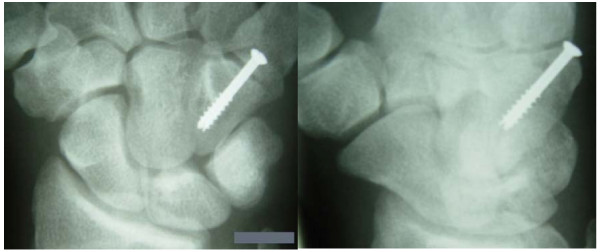
**Three month follow up anteroposterior (left) and oblique (right) x-ray views**.

Under sedation in theatre, further examination revealed 4^th ^ray carpo-metacarpal subluxation on stressing the joint indicating that this was a closed unstable injury. Open reduction and internal fixation of this fracture was successfully undertaken. Follow up at three months revealed a well maintained reduction of the fracture which was healed (Figure 3). At one year follow up the patient was pain free with a stable joint and a range of movement (ROM) of 0-90° which was consistent with ROM in other unaffected MCP joints.

## Discussion

The hamate bone is a roughly triangular-shaped bone, which is located in the distal carpal row farthest to the ulnar side. It is bordered proximally by the pisiform and the lunate in the proximal carpal row, radially by the capitate, and distally by the bases of the fourth and fifth metacarpals.

Hamate fractures are classified as either type I, involving the hook, or type II, involving the body, with type I fractures being more common. Hamate fractures are uncommon, particularly those involving the body of the hamate, however they are the commonest fracture of the distal carpal row [1] and are increasing in incidence possibly due to the increasing popularity of sports involving racquets, bats and clubs. They are associated with instability and unless detected and managed appropriately are associated with a poor outcome [2].

Traditionally, fractures and dislocation of the hamate are identified on AP or lateral x-ray views [3]. Carpal tunnel views and computed tomography (CT) [4,5] have also been suggested to help. This case highlights some of the problems encountered with traditional x-ray views, and the need to consider oblique views as either standard procedure or as an adjunct where clinical suspicion remains high even in the presence of normal x-rays. This point remains valid even given the increasing use and availability of other forms of radiological investigation [6-9].

We report the current case to highlight the following:

### 1. X-ray views

Andreson et al [1] concluded high resolution CT was the imaging modality of choice for body and hamate hook fractures. Their vitro experiments on 18 cadaver hands showed that CT had 100% sensitivity and 94.4% specificity and conventional X-ray showed 72.2% sensitivity and 88.8% specificity for detection of hamate fractures. However, our case demonstrates with supporting literature [1,2,10,11] the benefit of oblique views from 30 - 45° and these should be considered standard with anteroposterior, lateral and carpal tunnel views when hamate fracture is suspected. If detected with these, computerised tomography may be avoided.

### 2. Minimal palmar flexion injuries associated with carpal bone fractures

It is commonly recognised that hyperextension injuries to the hand are associated with carpal bone fractures, especially scaphoid. This case and others[2] establish a link between minimal palmar flexed injuries and hamate fractures.

We the authors believe that the 'standard' views for all wrist injuries should include;

• PA (posteroanterior)

• PA with ulnar flexion

• Medial oblique

• Lateral.

We also believe that in injuries where the hamate is thought to be involved OR where a high index of suspicion for bony injury remains in the presence of normal initial radiographs, that carpal tunnel views should be carried out. Furthermore several other oblique projections may be needed until the plane of the fracture is delineated clearly.

## Conclusion

The authors believe that where a hamate fracture is suspected an oblique x-ray view should be considered as part of the initial diagnostic investigations. It can help with diagnosis and give further important information to aid appropriate management. An oblique x-ray view is of particular use when clinical suspicion for hamate fracture remains high in the light of otherwise normal x-rays. Consideration and use of this view can negate the need for costly, time-consuming CT scans. We believe that the standard trauma series should be: PA; PA with ulnar flexion; medial oblique and lateral X-rays. With an additional carpal tunnel view where hamate fracture is suspected.

## Abbreviations

MCP: metacarpophalangeal; AP: anteroposterior; PA: posteroanterior; CT: computed tomography; ROM: range of movement.

## Consent

Written informed consent was obtained from the patient for publication of this case report and accompanying images. A copy of the written consent is available for review by the Editor-in-Chief of this journal.

## Competing interests

The authors declare that they have no competing interests.

## Authors' contributions

VB collected the information and wrote the report. JH assisted with the writing of the report and collected x-rays. AF had the initial idea for the report and is guarantor. All authors read and approved the final manuscript.
